# ‘The monthly curse’: menstrual-associated coronary artery vasospasm in a premenopausal woman—a case report

**DOI:** 10.1093/ehjcr/ytaf494

**Published:** 2025-10-03

**Authors:** Akari Odagi, Kensuke Matsumoto, Tetsu Yamazaki, Satoru Kawasaki, Hogara Nishisaki

**Affiliations:** Division of Cardiovascular Medicine, Department of Internal Medicine, Hyogo Prefectural Tamba Medical Centre, 2002-7, Isou, Hikami-cho, Tamba-shi, Hyogo, Tamba 669-3495, Japan; Division of Cardiovascular Medicine, Department of Internal Medicine, Hyogo Prefectural Tamba Medical Centre, 2002-7, Isou, Hikami-cho, Tamba-shi, Hyogo, Tamba 669-3495, Japan; Division of Cardiovascular Medicine, Department of Internal Medicine, Hyogo Prefectural Tamba Medical Centre, 2002-7, Isou, Hikami-cho, Tamba-shi, Hyogo, Tamba 669-3495, Japan; Division of Cardiovascular Medicine, Department of Internal Medicine, Hyogo Prefectural Tamba Medical Centre, 2002-7, Isou, Hikami-cho, Tamba-shi, Hyogo, Tamba 669-3495, Japan; Division of Cardiovascular Medicine, Department of Internal Medicine, Hyogo Prefectural Tamba Medical Centre, 2002-7, Isou, Hikami-cho, Tamba-shi, Hyogo, Tamba 669-3495, Japan

**Keywords:** Catamenial coronary vasospasm, Myocardial infarction with non-obstructive coronary arteries, Menstrual cycle, Oestrogen replacement therapy, Case report

## Abstract

**Background:**

Catamenial coronary vasospasm has recently been recognized as a refractory form of vasospastic angina, characterized by recurrent episodes of chest pain that are temporally associated with the menstrual cycle.

**Case summary:**

A 38-year-old premenopausal woman presented to our hospital with chest pain. Upon entering the cold examination room, she experienced chest pain identical in character to her previous episodes. Electrocardiography revealed ST-segment elevation in the inferior leads. Sublingual administration of nitroglycerin resulted in the prompt relief of symptoms and resolution of ST-segment changes. Subsequent coronary angiography revealed no obstructive coronary lesions, leading to the diagnosis of coronary vasospasm. A comprehensive clinical history revealed that all episodes of chest pain occurred consistently in the days preceding the onset of menstruation. She was closely monitored in an outpatient setting and was prescribed a combination of benidipine and nicorandil. Unfortunately, the patient was transferred to our hospital following an episode of cardiopulmonary arrest. Emergency coronary angiography revealed critical stenosis due to vasospasm in the ostium of the right coronary artery. The patient died despite receiving intensive care.

**Discussion:**

During the late luteal and menstrual phases, circulating oestrogen levels decline to their lowest point, potentially increasing susceptibility to coronary vasospasms. In recent years, oestrogen replacement therapy has been reported to confer therapeutic benefits in this patient population. Early multidisciplinary collaboration involving gynaecologists and timely initiation of hormonal therapy may alter the clinical course and prevent fatal outcomes in such patients.

Learning points

- Clinicians should maintain a high index of suspicion for catamenial coronary vasospasm in premenopausal women presenting with vasospastic angina.
- We actively need to explore the temporal association between anginal episodes and the menstrual cycle.
- Early multidisciplinary collaboration involving gynaecologists, along with the timely initiation of hormonal therapy, may favourably alter the clinical course and prevented the fatal outcomes in such patients.

## Introduction

Catamenial coronary vasospasm has recently been recognized as a rare and potentially refractory subtype of vasospastic angina characterized by recurrent episodes of chest pain temporally associated with the menstrual cycle in premenopausal women. Although sporadic and anecdotal cases have been documented in the literature,^1–6^ the true prevalence, pathophysiology, diagnostic criteria, and optimal management strategies remain poorly defined. This may be attributed to under-recognition and a lack of systematic investigation.

Herein, we describe a case of suspected catamenial coronary vasospasm in a premenopausal woman who experienced recurrent angina episodes despite being administered guideline-directed medical therapy and ultimately experienced a sudden fatal cardiac arrest.

## Summary figure

**Figure:**
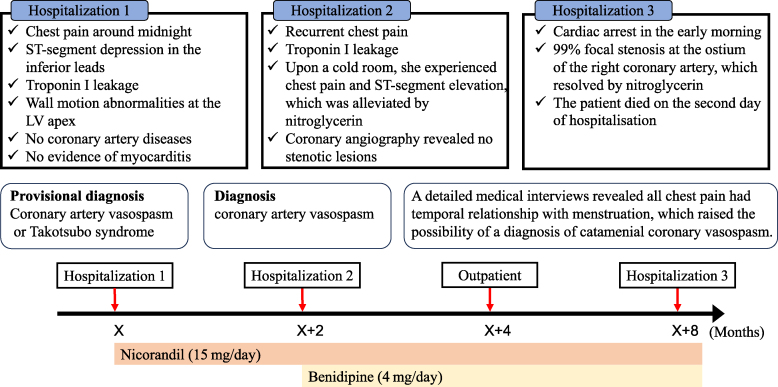


## Case presentation

**Figure 1:**
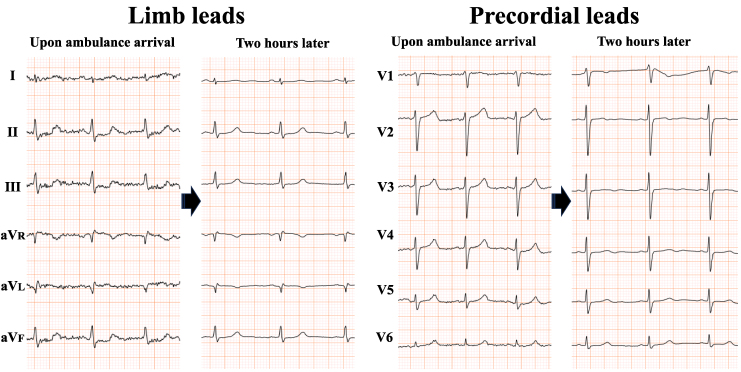
Electrocardiograms from a 38-year-old woman obtained upon ambulance arrival and again 2 h later. Initial electrocardiogram (left panels of both limb and precordial leads) displaying apparent ST-segment depression in leads II, III, and aVF. Two hours later, the abnormalities resolved spontaneously (right panels).

**Figure 2:**
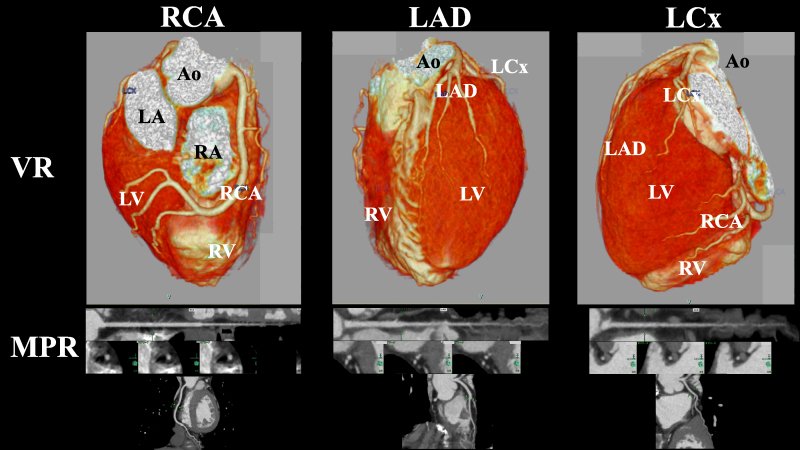
Multidetector-row computed tomography of a 38-year-old woman. Volume-rendering (upper panels) and multiplanar reconstruction images (lower panels) of the major epicardial coronary arteries from multidetector-row computed tomography revealed no evidence of coronary stenosis, thromboembolism, or coronary dissection. RCA, right coronary artery; LAD, left anterior descending artery; LCx, left circumflex artery; VR, volume rendering; MPR, multiplanar reconstruction; Ao, aorta; LV, left ventricle; LA, left atrium; RV, right ventricle; RA, right atrium.

**Figure 3:**
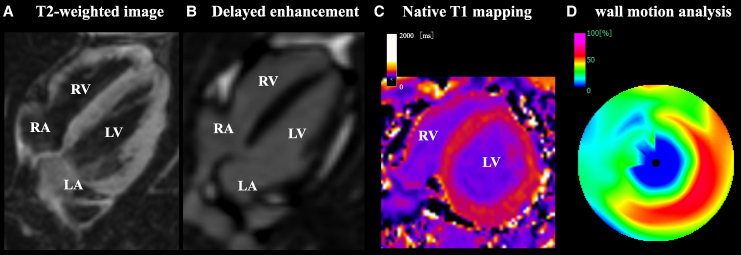
Gadolinium-enhanced cardiac magnetic resonance imaging of a 38-year-old woman. T2-weighted image (*A*), delayed gadolinium enhancement (*B*), native T1-mapping (*C*), and wall motion analysis (*D*) are presented, respectively. Findings suggestive of myocarditis, such as myocardial oedema, pericardial effusion, or late gadolinium enhancement indicative of myocardial scarring, were absent. Consistent with the echocardiography findings, regional wall motion abnormalities confined to the apical segments are noted.

A 38-year-old premenopausal woman presented with the sudden onset of chest discomfort occurring around midnight while attempting to use the bathroom. The patient was subsequently transported to our hospital. Her medical history was unremarkable for cardiovascular diseases and she had no prior history of syncope. Upon arrival, her vital signs were stable, with blood pressure of 128/89 mmHg, heart rate of 90 b.p.m., and oxygen saturation of 100% on ambient air. Although the physical examination was normal, the patient exhibited hyperventilation, with a respiratory rate of 30 breaths per minute. The initial electrocardiogram (ECG) demonstrated ST-segment depression in leads II, III, and aVF. However, her symptoms resolved spontaneously, with concurrent resolution of the ECG abnormalities (*Figure 1*). Laboratory findings on admission revealed a mildly elevated troponin I level of 52.1 pg/mL (reference < 26.2 pg/mL), which increased markedly to 945.7 pg/mL within 2 h. Transthoracic echocardiography demonstrated preserved left ventricular (LV) systolic function with an ejection fraction of 64.8% (*Video 1*), along with regional wall motion abnormalities localized to the LV apex (*Video 2*). Multidetector-row computed tomography excluded obstructive coronary artery disease, coronary thromboembolism, and dissection (*Figure 2*). Based on these findings, myocardial infarction due to coronary artery disease was effectively ruled out, and cardiac magnetic resonance imaging with gadolinium contrast was performed. Magnetic resonance imaging confirmed apical wall motion abnormalities consistent with those observed on echocardiography but revealed no evidence of myocarditis, specifically, no myocardial oedema, pericardial effusion, or late gadolinium enhancement indicating fibrosis (*Figure 3*). These comprehensive multimodal investigations culminated in a provisional diagnosis of either coronary artery vasospasm or Takotsubo syndrome. Following the normalization of troponin levels, the patient was discharged on Day 3 with nicorandil (15 mg/day) initiated as a coronary vasodilator. Because there was no history of syncope, an implantable cardiac monitoring device was not implanted.

**Figure 4:**
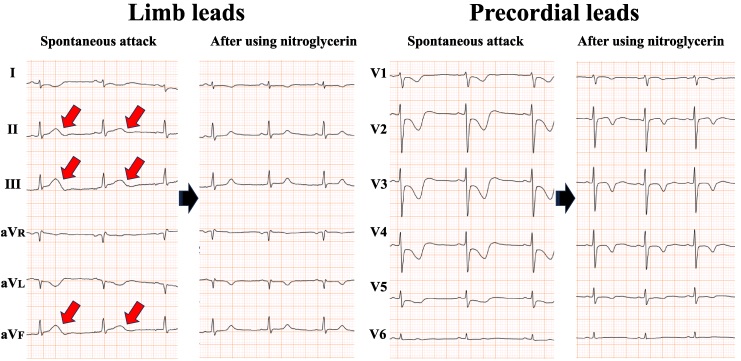
Electrocardiograms of a 38-year-old woman obtained during spontaneous attack and following administration of nitroglycerin. Electrocardiography during a spontaneous attack displaying ST-segment elevation in the inferior leads, accompanied by reciprocal changes in the precordial leads (left panels). Following the administration of two puffs of sublingual nitroglycerin spray, the patient’s symptoms rapidly improved, and the ST-segment elevation resolved accordingly (right panels). LV, left ventricle; LA, left atrium; RV, right ventricle; RA, right atrium.

**Figure 5:**
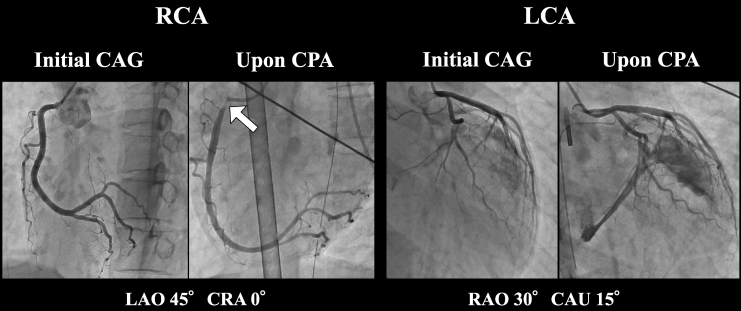
Coronary angiograms of a 38-year-old woman obtained during second hospitalization and at the time of cardiopulmonary arrest. Coronary angiography performed during the second hospitalization reveals no organic stenotic lesions (left panel). In contrast, 99% stenosis of the ostium of the right coronary artery is observed at the time of cardiopulmonary arrest (right panel). RCA, right coronary artery; LCA, left coronary artery; CAG, coronary angiography; CPA, cardiopulmonary arrest; LAO, left anterior oblique; RAO, right anterior oblique; CRA, cranial; CAU, caudal.

Two months later, the patient returned to our outpatient clinic complaining of recurrent chest pain. Her troponin I level was mildly elevated (66.0 pg/mL), prompting readmission with suspected acute coronary syndrome. A hyperventilation provocation test was scheduled for the second day of hospitalization. Upon entering a cold examination room with strong air conditioning, she immediately experienced chest pain identical to her previous episodes. Electrocardiogram demonstrated ST-segment elevation in the inferior leads with reciprocal changes in the precordial leads (*Figure 4*, left panels). Sublingual administration of nitroglycerin spray resulted in the prompt relief of symptoms and rapid resolution of ST-segment changes (*Figure 4*, right panels). Coronary angiography revealed no obstructive coronary lesions (*Figure 5*, left panels). A definitive diagnosis of coronary vasospasm was made based on the standard diagnostic criteria.^7^ Detailed clinical history elicited that all chest pain episodes consistently preceded the onset of menstruation. Based on this temporal relationship, the rare diagnosis of catamenial coronary vasospasm was considered. Despite isosorbide mononitrate was prescribed, the patient developed headaches as a side effect. Thus, a combination therapy of nicorandil (15 mg/day) and benidipine (4 mg/day) was selected as the alternative treatment. After the second discharge, she was followed up on outpatient basis every 2 months. She continued aforementioned combination therapy, during which time her symptoms remained well controlled. However, on one occasion, she experienced an episode of chest oppression in the premenstrual period, which resolved immediately after administration of sublingual nitroglycerin spray. We had actually planned to consult a gynaecologist promptly when angina attack shows signs of destabilization.

Unfortunately, 6 months following her second discharge, the patient was discovered unresponsive in the early morning hours and was urgently transported to our hospital in cardiopulmonary arrest. Mechanical circulatory support was initiated with venoarterial extracorporeal membrane oxygenation. Emergency coronary angiography revealed 99% focal stenosis at the ostium of the right coronary artery (*Figure 5*), which completely resolved following intracoronary nitroglycerin administration. These findings are indicative of critical coronary vasospasm as the precipitating event leading to cardiac arrest. Despite receiving intensive care, the patient died on the second day of hospitalization. Although a post-mortem autopsy was requested, we were unable to obtain the family’s consent.

## Discussion

Myocardial infarction with non-obstructive coronary arteries is defined as a clinical syndrome characterized by the occurrence of myocardial infarction in the absence of significant obstructive lesions in the epicardial coronary arteries.^8^ This heterogeneous syndrome encompasses various underlying pathophysiological mechanisms, among which coronary artery vasospasm is recognized as one of the most prevalent causes.^8^ Although coronary vasospasm is more frequently observed in Asian populations, its occurrence in premenopausal women is rare.^9^ Moreover, the condition is generally responsive to pharmacological therapy, with life-threatening or refractory cases considered rare exceptionas.^9^ In recent years, however, increasing attention has been directed towards a specific subset of treatment-resistant coronary vasospasm that appears to occur in temporal association with particular phases of the menstrual cycle. A decline in circulating oestrogen levels has been postulated to contribute to the onset of coronary vasospasm in susceptible individuals.^2,10^

Oestrogen has been demonstrated to increase endothelial nitric oxide synthase activity, thereby promoting vasodilation.^10^ During the late luteal and menstrual phases, circulating oestrogen levels decline to their nadir, potentially reducing vasodilatory capacity and thereby predisposing to coronary vasospasm.^11^ Taken together, epidemiological observations,^7–9^ mechanistic insights,^2^ and case-based evidence^1,4,5^ support the possibility that oestrogen replacement therapy offers therapeutic benefits in this distinct population. Kawano *et al*.^2^ evaluated the efficacy of oestrogen therapy in 15 women with vasospastic angina through serial hyperventilation provocation testing conducted before, during, and after hormone administration. Anginal episodes were reproducibly provoked both before and after cessation of oestrogen therapy but were absent during treatment. Furthermore, Tezuka *et al*.^1^ reported the complete suppression of anginal attacks in a young woman with refractory catamenial coronary vasospasm following hormone replacement therapy. In light of these findings, oestrogen therapy has garnered attention as a potentially effective treatment modality for catamenial coronary vasospasm.

In retrospect, provocation testing should have been undertaken following the initiation of anti-anginal therapy, with the aim of evaluating not only therapeutic efficacy but also refractoriness to the therapy.^12^ Moreover, had invasive coronary physiological assessment been performed and revealed microvascular dysfunction, a more aggressive therapeutic strategy might have been introduced.^13^ Such a clinical approach might have prevented an underestimation of both disease severity and refractoriness to the initial regimen and could have facilitated the timely implementation of a more intensive therapeutic strategy. Moreover, if hormone levels had been measured, it may have strengthened the physiological relevance of oestrogen decline, leading to more aggressive treatment strategies. These included early multidisciplinary collaboration involving gynaecologists, the timely initiation of hormonal therapy, and the addition of the denopamine for refractory vasospastic angina,^12^ which may have altered the clinical course. On the other hand, not only has the threshold of oestrogen level capable of inducing catamenial coronary vasospasm not yet been determined, but the patient characteristics predisposing to this condition also remain unknown. We believe that bridging this knowledge gap will require the collection of data from a larger number of cases, accompanied by detailed analysis.

## Conclusion

Catamenial coronary vasospasm remains an under-recognized yet potentially life-threatening clinical entity. In premenopausal women presenting with vasospastic angina, clinicians should maintain a high index of suspicion for catamenial coronary vasospasm and actively assess for a temporal association between anginal episodes and the menstrual cycle. Increased clinical awareness, timely diagnostic evaluation, and early multidisciplinary collaboration are essential for optimal management. Prompt initiation of appropriate therapy, including hormonal modulation, may be the key to preventing catastrophic outcomes.

## Lead author biography


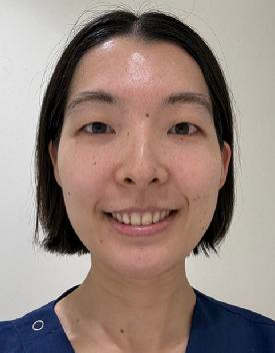


Akari Odagi graduated from Kobe University, Japan, in 2022. After completing junior residency at Hyogo Prefectural Tamba Medical Centre, she is currently an internal medicine resident with a major in cardiology at the same institution. Her interest is in intensive cardiac care and structural heart diseases.

## Data Availability

Data are accessible upon reasonable request; however, the authors reserve the right to individually assess and decide upon each request.
